# The Monarch Initiative in 2019: an integrative data and analytic platform connecting phenotypes to genotypes across species

**DOI:** 10.1093/nar/gkz997

**Published:** 2019-11-08

**Authors:** Kent A Shefchek, Nomi L Harris, Michael Gargano, Nicolas Matentzoglu, Deepak Unni, Matthew Brush, Daniel Keith, Tom Conlin, Nicole Vasilevsky, Xingmin Aaron Zhang, James P Balhoff, Larry Babb, Susan M Bello, Hannah Blau, Yvonne Bradford, Seth Carbon, Leigh Carmody, Lauren E Chan, Valentina Cipriani, Alayne Cuzick, Maria Della Rocca, Nathan Dunn, Shahim Essaid, Petra Fey, Chris Grove, Jean-Phillipe Gourdine, Ada Hamosh, Midori Harris, Ingo Helbig, Maureen Hoatlin, Marcin Joachimiak, Simon Jupp, Kenneth B Lett, Suzanna E Lewis, Craig McNamara, Zoë M Pendlington, Clare Pilgrim, Tim Putman, Vida Ravanmehr, Justin Reese, Erin Riggs, Sofia Robb, Paola Roncaglia, James Seager, Erik Segerdell, Morgan Similuk, Andrea L Storm, Courtney Thaxon, Anne Thessen, Julius O B Jacobsen, Julie A McMurry, Tudor Groza, Sebastian Köhler, Damian Smedley, Peter N Robinson, Christopher J Mungall, Melissa A Haendel, Monica C Munoz-Torres, David Osumi-Sutherland

**Affiliations:** 1 Center for Genome Research and Biocomputing, Environmental and Molecular Toxicology, Oregon State University, Corvallis, OR 97331, USA; 2 Environmental Genomics and Systems Biology, Lawrence Berkeley National Laboratory, Berkeley, CA 94710, USA; 3 The Jackson Laboratory For Genomic Medicine, Farmington, CT 06032, USA; 4 European Bioinformatics Institute (EMBL-EBI), European Molecular Biology Laboratory, Wellcome Genome Campus, Hinxton, Cambridge CB10 1SD, UK; 5 Oregon Clinical and Translational Research Institute, Oregon Health & Science University, Portland, OR 97239, USA; 6 Renaissance Computing Institute at UNC, Chapel Hill, NC 27517, USA; 7 Broad Institute, Cambridge, MA 02142, USA; 8 The Jackson Laboratory, Bar Harbor, ME 04609, USA; 9 Institute of Neuroscience, University of Oregon, Eugene, OR 97401, USA; 10 College of Public Health and Human Sciences, Oregon State University, Corvallis, OR 97331, USA; 11 William Harvey Research Institute, Barts & The London School of Medicine & Dentistry, Queen Mary University of London, Charterhouse Square, London EC1M 6BQ, UK; 12 Rothamsted Research, Harpenden AL5 2JQ, UK; 13 Office of Rare Diseases Research (ORDR), National Center for Advancing Translational Sciences (NCATS), National Institutes of Health (NIH), Bethesda, MD 20892, USA; 14 dictyBase, Center for Genetic Medicine, Northwestern University, Chicago, IL 60611, USA; 15 California Institute of Technology, Pasadena, CA 91125, USA; 16 McKusick-Nathans Institute of Genetic Medicine, Johns Hopkins University, Baltimore, MD 21205, USA; 17 University of Cambridge, Cambridge CB2 1TN, UK; 18 Division of Neurology, Children's Hospital of Philadelphia, Philadelphia, PA 19104, USA; 19 Department of Biomedical and Health Informatics, Children's Hospital of Philadelphia, Philadelphia, PA 19104, USA; 20 Department of Neuropediatrics, Christian-Albrechts-University of Kiel, 24105 Kiel, Germany; 21 Department of Neurology, University of Pennsylvania, Perelman School of Medicine, Philadelphia, PA 19104, USA; 22 Department of Biochemistry and Molecular Biology, Oregon Health & Science University, Portland, OR 97239, USA; 23 Pryzm Health, 4215 Queensland, Australia; 24 Autism & Developmental Medicine Institute, Geisinger, Danville, PA 17837, USA; 25 Stowers Institute for Medical Research, Kansas City, MO 64110, USA; 26 Xenbase, Cincinnati Children's Hospital, Cincinnati, OH 45229, USA; 27 National Institute of Allergy and Infectious Diseases, National Institutes of Health, Bethesda, MD 20892, USA; 28 University of North Carolina Medical School, University of North Carolina at Chapel Hill, Chapel Hill, NC 27516, USA; 29 Institute for Medical Genetics and Human Genetics, Charité-Universitätsmedizin Berlin, Augustenburger Platz 1, 13353 Berlin, Germany

## Abstract

In biology and biomedicine, relating phenotypic outcomes with genetic variation and environmental factors remains a challenge: patient phenotypes may not match known diseases, candidate variants may be in genes that haven’t been characterized, research organisms may not recapitulate human or veterinary diseases, environmental factors affecting disease outcomes are unknown or undocumented, and many resources must be queried to find potentially significant phenotypic associations. The Monarch Initiative (https://monarchinitiative.org) integrates information on genes, variants, genotypes, phenotypes and diseases in a variety of species, and allows powerful ontology-based search. We develop many widely adopted ontologies that together enable sophisticated computational analysis, mechanistic discovery and diagnostics of Mendelian diseases. Our algorithms and tools are widely used to identify animal models of human disease through phenotypic similarity, for differential diagnostics and to facilitate translational research. Launched in 2015, Monarch has grown with regards to data (new organisms, more sources, better modeling); new API and standards; ontologies (new Mondo unified disease ontology, improvements to ontologies such as HPO and uPheno); user interface (a redesigned website); and community development. Monarch data, algorithms and tools are being used and extended by resources such as GA4GH and NCATS Translator, among others, to aid mechanistic discovery and diagnostics.

## INTRODUCTION

The quest to elucidate the genetic basis of disease is hampered by the fragmented landscape of clinical and organismal data. The Monarch Initiative is an open-source resource that has amassed a large collection of genotype–phenotype data: over two million phenotypic associations from dozens of sources covering over 100 species. Monarch provides a bridge between basic and clinical research, developing tools to connect data from multiple sources using ontologies and semantic data integration. Over the past three years, we have introduced and extended numerous integrated ontologies for disease, phenotype, genotype and anatomy, to enable deep semantic integration and cross-species querying. Monarch's data resources, APIs and analysis tools are used both internally and by external groups to bring the power of research organismal data to the clinical domain. Monarch has pioneered the use of research organism data for rare disease diagnosis, and a number of our resources have become global standards.

## PRODUCTS OF THE MONARCH INITIATIVE

### Ontologies

Monarch develops methods and tools that not only support precision medicine and disease modeling, but also support mechanistic exploration of the relationships between genotype, environment and phenotype across the tree of life. We do this by using ontologies to leverage semantic relationships between biological concepts. Members of the Monarch Initiative and our collaborators have developed key ontological resources such as the Human Phenotype Ontology (HPO); have employed innovative techniques to harmonize phenotype ontologies in the Unified Phenotype Ontology (uPheno); have similarly harmonized disease terminologies in the creation of Mondo; and continue to develop these and other resources that work together to build toward an interoperable semantic landscape. Below, we describe our latest work on ontologies.

#### The Human Phenotype Ontology (HPO)

For proper semantic representation of clinical abnormalities and its associations to diseases we developed the HPO. HPO (https://hpo.jax.org), a flagship of Monarch, is a standardized vocabulary of phenotypic abnormalities associated with over 7000 diseases (1). HPO is the *de facto* standard terminology for clinical ‘deep phenotyping’ in humans, providing detailed descriptions of clinical abnormalities and computable disease definitions. This ontology enables non-exact matching of sets of phenotypic features (phenotype profiles) against known diseases, other patients and research organisms. The primary labels in the HPO employ medical terminology used by clinicians and researchers. To make the HPO more accessible to patients and non-medical experts, we added layperson synonyms, where appropriate (2). The HPO currently contains 4887 terms with at least one lay synonym, and these are available via the HPO website or in the OWL file with the ‘layperson term’ subset tag.

Algorithms based on HPO have been implemented in many diagnostic and variant prioritization tools and are used by the UK’s 100 000 Genomes Project (3), the US NIH Undiagnosed Diseases Program (4) and Network (5), and thousands of other clinics, labs, tools, and databases worldwide. We have developed strong ties with clinical adopters to continue improving specific areas of the ontology and extend standardized disease descriptions. Initially developed for rare disease phenotyping, HPO also captures many phenotypes for common diseases and can be used as a general resource for patient phenotyping. We continue to explore patient phenotyping from other sources of electronic health records (EHRs) and to promote the adoption of HPO in health care systems (6).

#### The Unified Phenotype Ontology (uPheno)

Different ontologies are used to represent the phenotypes in humans and in each of the major model organism groups. For example, the Mammalian Phenotype (MP) ontology (7) is used by the Mouse Genome Informatics resource (MGI) (8) to annotate mouse phenotypes, HPO by Monarch for human phenotypes and the Drosophila Phenotype Ontology (9) by FlyBase for *Drosophila* Phenotypes. The Zebrafish Information Network (ZFIN) (10) uses a different approach, describing zebrafish phenotypes with terms from reference ontologies such as the Phenotype And Trait Ontology (PATO; http://www.obofoundry.org/ontology/pato) and the Gene Ontology (GO) (11). To enable cross-species querying of phenotypes we have developed an approach for integrating these ontologies based on logical definitions (12), called the Entity-Quality (EQ) approach. In 2013 we implemented this approach in the Unified Phenotype Ontology (uPheno) that integrates organism-specific phenotype ontologies using ‘bridging axioms' that connect terms from different ontologies using equivalence or subsumption axioms (13).

**Figure 1.**
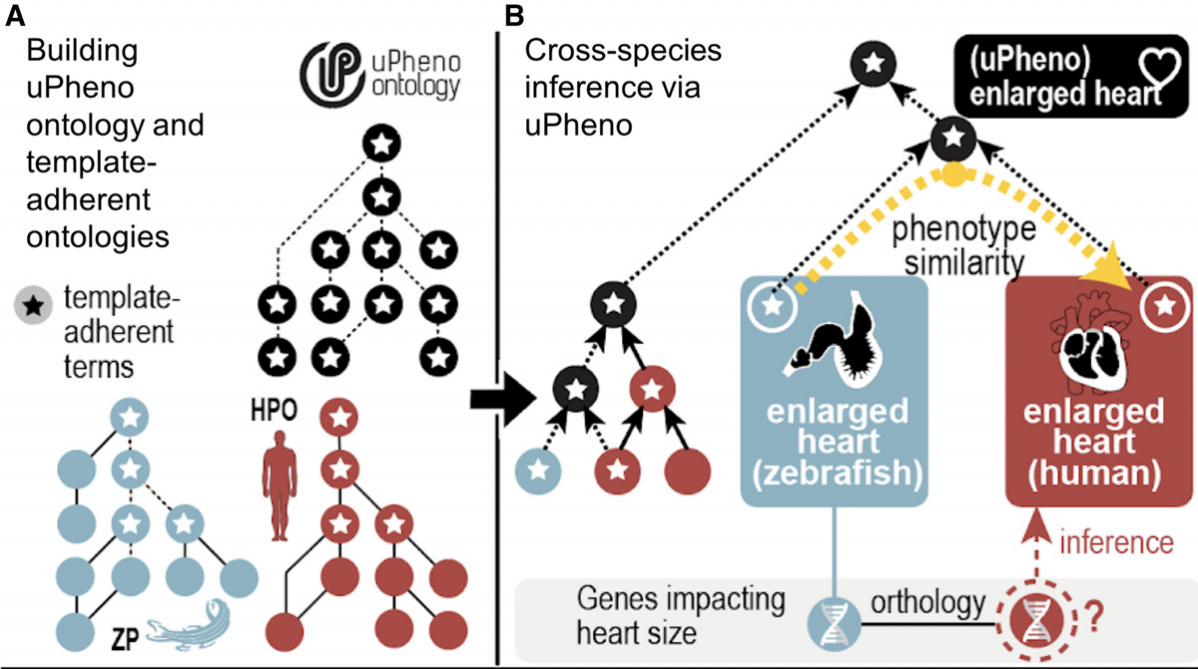
uPheno template-driven ontology development and harmonization. uPheno templates are used to define phenotypes according to agreed upon design patterns. (**A**). Computable definitions specified using uPheno templates are used to automate classification of uPheno and parts of the Zebrafish Phenotype Ontology (ZP (13); dashed lines). (**B**). Computable definitions also drive automated classification of HPO and ZP classes under uPheno classes. For example, enlarged heart in ZP (defined using the zebrafish anatomy heart term) and enlarged heart in HPO are both classified under uPheno enlarged heart (defined using Uberon heart). Algorithms can use this classification under uPheno to predict that human orthologs of zebrafish genes annotated to enlarged heart may cause enlarged heart in humans.

The Monarch Initiative uses uPheno (http://obofoundry.org/ontology/upheno) to find candidate genes and potential animal models for human diseases. For this to work well, similar terms both within and between phenotype ontologies need to be described in a logically consistent manner (14). However, while the EQ approach was widely adopted by important model organism communities such ZFIN (10), MGI (8), WormBase (15) and FlyBase (16), the logical definitions themselves were developed in relative isolation, which often caused them to be semantically incompatible. In 2018, we launched a community-wide effort to reconcile and align phenotype ontologies (17) which defined templates based on the Dead Simple Ontology Design Pattern (DOSDP) (18) framework (Figure 1) for the consistent and interoperable representation of phenotypes (for more details on the Reconciliation Effort see section ‘Community Engagement’). Based on these patterns, we developed a novel framework (https://github.com/obophenotype/upheno-dev) for the automated construction of uPheno (https://f1000research.com/posters/8-403). We are currently working closely with members from 14 phenotype ontologies and databases to expand the coverage of species-specific phenotypes in uPheno with organisms including *C.elegans*, *Xenopus*, *Dictyostelium discoideum*, *Schizosaccharomyces pombe* and more. This work advances a core mission of the Monarch Initiative: to leverage the wealth of phenotypic knowledge generated by the study of multiple species to understand the genetic nature of human disease.

#### Mondo: The unified disease ontology

Mondo (http://obofoundry.org/ontology/mondo) is an ontology of diseases and disorders with over 23 000 terms describing a variety of diseases spanning Mendelian, rare, common, complex, infectious and cancer. We created Mondo by integrating available knowledge sources, defining which terms are truly equivalent across different resources. The result thereby enables the integration of associated information, such as treatments, genetics and phenotypes for diagnostics and mechanism discovery. There are many disease terminologies (19), with terms that typically cross-reference each other in ambiguous and conflicting ways. Mondo combines disease information from sources such as OMIM (20), Orphanet (21) and NCIt (National Cancer Institute Thesaurus (22)), in order to leverage the strengths of each resource, including the neoplastic disease classification of NCIt, the rare disease coverage of Orphanet, the Mendelian coverage of OMIM and the common disease coverage of other resources. The Mondo build process uses novel, scalable computational methods to find, untangle and resolve conflicts occurring when disease nomenclatures are merged using cross-references. As a result, Mondo is a logically coherent, merged disease ontology, and it constitutes a scalable solution to the challenge of integrating multiple, partially overlapping and partially conflicting disease terminologies. The Mondo ontology is used in a growing number of bioinformatics resources, including ClinGen (23), the Genetic and Rare Diseases Information Center (GARD, https://rarediseases.info.nih.gov), the European Bioinformatics Institute (EMBL-EBI) as a component of the Experimental Factor Ontology (EFO) (24) and the Kids First Data Resource Portal (https://kidsfirstdrc.org). Initially constructed using semi-automatic methods (25), Mondo is now extensively manually curated. A new release for Mondo is available monthly.

#### SEPIO

Monarch developed the SEPIO ontology-based data modeling framework for representing evidence and provenance behind scientific assertions (http://obofoundry.org/ontology/sepio). SEPIO stands for Scientific Evidence and Provenance Information Ontology. Initially developed to support harmonized representation of the diverse evidence and provenance information across knowledge sources integrated by Monarch, SEPIO has since been adopted and expanded by external efforts. For example, the ClinGen consortium is defining SEPIO-based data models for all five of its curation pipelines, including a Variant Pathogenicity Interpretation data model aligned with the 2015 ACMG Guidelines (https://dataexchange.clinicalgenome.org/interpretation). Several other projects are in the process of defining SEPIO-based data models, including the Variant Interpretation in Cancer Consortium (VICC, https://cancervariants.org), and the GA4GH Genomic Knowledge Standards working group.

#### GENO

**Figure 2.**
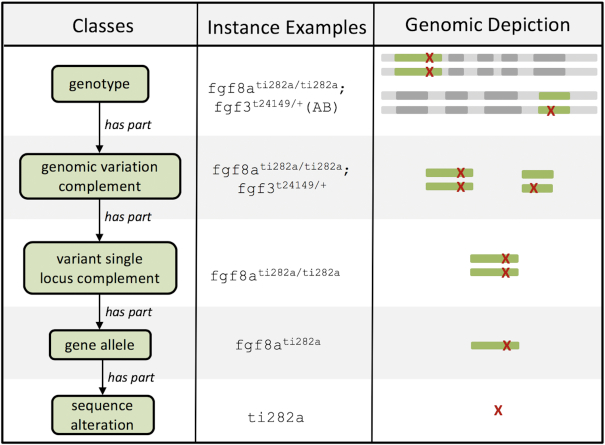
Decomposition of a Zebrafish Genotype. The left panel shows classes in the core genotype partonomy. The center panel shows an example instance of each class from the zebrafish genotype (see also https://zfin.org/ZDB-GENO-161227-1). The right panel shows a graphical depiction of the portion of the genome specified at each level (where the top panel shows a complete genome composed of two sets of homologous chromosomes).

The Genotype Ontology, GENO (http://obofoundry.org/ontology/geno), is an ontology that represents the various components of genotypes, their relationships, and their characteristics. Figure 2 shows classes in the core GENO partonomy (e.g. transitive parts), which decompose a complete genotype into smaller components that reflect the levels at which phenotype annotations are made in different genotype–phenotype resources. For example, ZFIN and MGI annotate full zebrafish and mouse genotypes, respectively, while WormBase annotates gene alleles, and ClinVar (26) annotates individual human sequence alterations (e.g. a single nucleotide variation). The logic encoded in the GENO ontology allows inference of phenotype associations in a way that enables integrated analysis of data across knowledge sources and species. For example, a phenotype annotation made on the zebrafish genotype ‘fgf3^t24149/+^(AB)’ can be propagated down the partonomy to the ‘fgf3^t24149^’ gene allele, allowing direct comparison with data from sources such as WormBase that annotate phenotypes directly on gene alleles.

We have recently evolved GENO to accommodate new use cases from various community partners. For example, we have coordinated with the ClinGen Data Exchange Working Group (http://dataexchange.clinicalgenome.org) to add terms describing copy number variation, allelic phase, allele origin, and allelic state. We are also working with the Alliance of Genome Resources (https://www.alliancegenome.org) to align core GENO terms with genotype-related concepts used in the model organism community. These efforts will facilitate use of GENO to integrate data across a broader set of resources.

#### The integrated Monarch ontology

Beyond the ontologies developed by the Monarch Initiative, the Monarch platform brings together more than 30 ontologies including GO, the Cell Ontology (CL) (27), Uberon (28) and a number of organism-specific anatomy ontologies, such as FBbt (Drosophila) (https://wiki.flybase.org/mediawiki/images/a/a1/ISB2019_DAO.pdf) and ZFA (zebrafish) (29). To leverage these diverse ontologies in our knowledge graph and in the Monarch app, we have developed the Integrated Monarch Ontology (https://github.com/monarch-initiative/monarch-ontology). One of the key goals of the Monarch Ontology is to ensure interoperability between the integrated source ontologies which often depend on incompatible versions of other ontologies. To that end, we are currently developing a modular strategy based on the Ontology Development Kit (ODK, https://github.com/INCATools/ontology-development-kit). This strategy involves the integration and community wide deployment of so-called ‘base’ modules—subsets of the ontologies that contain only the native axioms (axioms actually belonging to the ontology) and exclude axioms from their respective ontology dependencies. This ensures that only the latest versions of all ontologies make it into the knowledge graph and stale and possibly incompatible dependencies on old versions are excluded. The Monarch Ontology forms the upper ontological layer of our knowledge and data graph, which will be described in the following sections.

## THE MONARCH ARCHITECTURE AND API

### Monarch architecture

**Figure 3.**
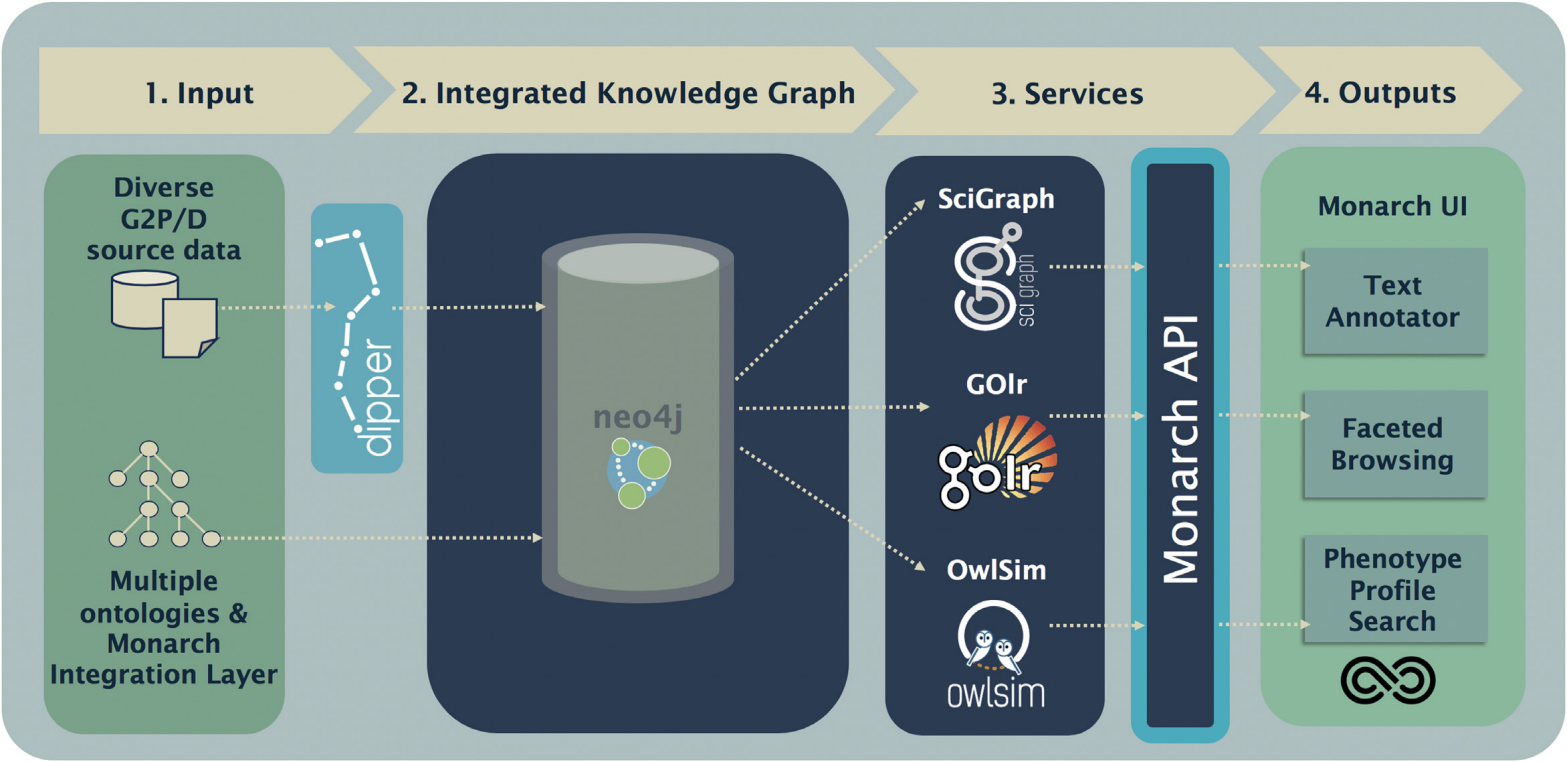
A workflow diagram of the Monarch architecture. Since our last report, we have developed the Monarch API (highlighted) for accessing associations between entities, performing computations on phenotype profiles, executing graph traversal queries, and performing text annotation (https://api.monarchinitiative.org/api).

As previously reported, we developed an ETL (Extract, Transform, Load) pipeline called Dipper (https://github.com/monarch-initiative/dipper), which ingests a wide range of data sources, including genes, mechanisms and context, as well as phenotypic and disease data from disparate sources including research organism and human databases. After ingestion, these data sources are regularized to conform to common association patterns according to the BioLink Model (https://biolink.github.io/biolink-model), are augmented with terms from many ontologies, and are published individually in the Resource Description Framework (RDF) format at https://archive.monarchinitiative.org/latest. These intermediate RDF data files, as well as all referenced ontologies, are unified with SciGraph (https://github.com/SciGraph/SciGraph), an application that wraps a Neo4j graph database and manages the transformation of ontologies and data described using ontologies into a combined graph which is henceforth referred to as the Monarch knowledge graph. This graph database serves as the primary data store for Monarch and its applications. We query and cache results from this graph database, indexing them with an ontology-enhanced Solr instance called GOlr, to provide quick access via our Monarch API, an OpenAPI-compliant data access layer (Figure 3).

### Data integration

**Figure 4.**
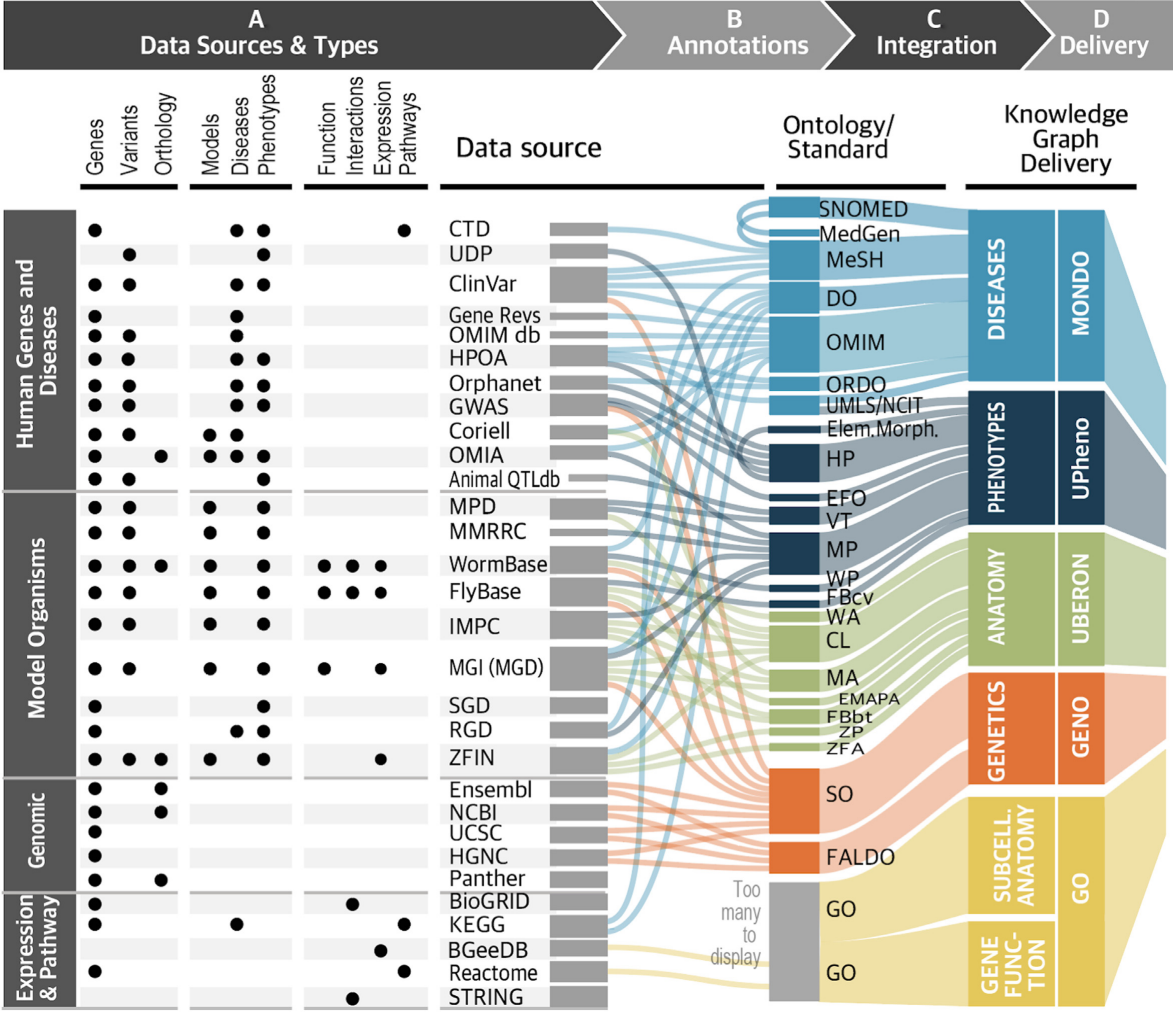
Monarch's data sources. The leftmost set of columns shows the types of data that the integrated data sources serve to Monarch. Note that these sources offer many additional data types that have not yet been integrated into Monarch. Each data source is annotated to specific ontologies and standards, which are, in turn, harmonized using the ontologies indicated in the rightmost panel. Those are used to create an integrated knowledge graph which drives the views and analytics on the Monarch website.

We regularly update our database with the latest gene-to-phenotype data from research organism databases (e.g. MGD (8), ZFIN (10), WormBase (15), FlyBase (16), IMPC (30)), human variants and gene-to-disease data (from OMIM, ClinVar, Orphanet, GWAS Catalog (31)) and other organismal gene-to-phenotype resources (OMIA (32), Animal QTLdb (33)). As well, we ingest other genomic data types, such as GO annotations, gene expression in specific tissues (BgeeDB (34)), protein-to-protein interaction (BioGRID (35)), pathway data (KEGG (36), Reactome (37)), chemical-disease associations (CTD (38)), cell line genotypes-to-disease data (Coriell; https://www.coriell.org), data from the Mouse Phenome Database (39) and from the Mutant Mouse Resource and Research Centers (MMRRC; https://www.mmrrc.org/, Office of the Director grant number OD010921). We recently also added data from the Rat Genome Database (RGD (40)), the Saccharomyces Genome Database (SGD (41)) and data from protein-to-protein interaction networks from STRING (42). The latest release of the Monarch knowledge graph (September 2019, https://archive.monarchinitiative.org/latest) contains over 32.9 million nodes and 160 million edges. In comparison to our previous report, we have 134 244 additional gene-to-phenotype associations. Nearly half (68 640) of the new associations were the result of adding SGD and RGD as new sources of data for Monarch, while 65 604 were added from new data available for mouse, zebrafish, nematode and human combined. Our database now has 26 433 models of disease, a 44% increase since our 2017 report in NAR (43). There are 2 982 400 high-quality protein-protein interactions from STRING from 6 species, and 931 518 from BioGRID. Figure 4 summarizes the sources, their data types, the ontologies used for integration and their delivery within Monarch's knowledge graph.

### Gene-to-disease and variant-to-disease data

In order to obtain a fine-grained resolution for genotype–phenotype relationships in humans, Monarch ingests variant-level data and gene-to-disease associations from OMIM, Orphanet, ClinVar and GWAS Catalog. Each source defines specific relationships between genes, variants and diseases depending on different forms of supporting evidence and other factors. Taken together, these sources contain information about 13 different relationships, some directly comparable and some not. Monarch uses these 13 relationships to link genes and variants to diseases, a subset of which is reserved for causal associations between a variation and a disorder, while others are utilized for non-causal associations, such as susceptibility to complex diseases, genome-wide association studies and variants that are likely pathogenic for a condition. The two levels of associations are shown separately in the results tables on our website, and can be queried separately using our API. Our latest data release shows that 3799 protein coding genes are identified as being the main cause for one or more disorders, as opposed to gene variants that contribute to disease susceptibility.

### Monarch application programming interface (API)

The Monarch API is a data access layer that sits on top of Monarch's knowledge graph and provides a standard way to access information about entities and association between entities, perform ontology navigation, run semantic similarity queries, perform annotation sufficiency scoring and annotate text with entities via named-entity recognition. Our API has a Swagger-generated documentation interface (https://api.monarchinitiative.org/api/swagger.json) that details all routes available for querying the Monarch database, including required and optional input parameters, JSON schema for the response, and working examples to guide users as they explore each API endpoint. Entity search supports labels, synonyms (e.g. layperson) and definitions. Search has been updated to include genotypes, variants and anatomical entities. All Monarch user interface components are driven by this API (i.e. search, data tables and text annotation). This unified data access layer enables researchers and physicians to explore the Monarch data on our website, and allow us to: (i) run phenotypic comparative analysis making use of semantic similarity software; (ii) annotate text; and (iii) browse data using facets–for example, disease or phenotype categories such as cardiovascular abnormalities (e.g. https://monarchinitiative.org/phenotype/HP:0001626).

In order to simplify programmatic access to Monarch's resources we developed Ontobio, a library written in Python and designed for work with ontologies and ontology associations. It supports a wide range of functionalities such as (i) parsing and using ontologies; (ii) an object model for working with ontologies and their metadata elements; (iii) an API for performing graph operations traversing through an ontology; (iv) ways to access associations from Monarch's knowledge graph; (v) ways to access functional annotations from the Gene Ontology; (vi) tools for performing enrichment analyses with virtually any ontology and associations for that ontology; and (vii) a command-line interface. Ontobio is freely available at https://github.com/biolink/ontobio. As part of this library, we also provide examples of how to use Ontobio for performing the aforementioned functionalities. Our API makes use of the Ontobio library for communicating with Monarch resources, enabling the Monarch API to remain a lightweight data access layer.

## THE NEW MONARCH USER INTERFACE

Monarch will soon be releasing a new, redesigned web interface for Monarch users. The new website presents users with a simplified format for accessing and exploring our data. With the semantic tools available from the Monarch Initiative, researchers, clinicians and the general public can gather, collate and unify disease information across human, research organisms and veterinary species in a single platform. A beta version of the updated user interface (UI) is available at https://beta.monarchinitiative.org. The new UI is a single page application, written in VueJS, that relies on various backend services, primarily our new API (described above), to retrieve and display the data. The Monarch database integrates information from 31 phenotype-related resources (https://beta.monarchinitiative.org/about/data-sources), allowing users to establish connections among biological entities of interest, such as genes, genotypes, gene variants (including SNPs, SNVs, QTLs, CNVs), models (including cell lines, animal strains, species, breeds, as well as targeted mutants), pathways, orthologs, phenotypes and publications. From the new home page, users will be able to explore Monarch using names or identifiers for phenotypes, diseases, genes, publications, variants or models of human disease. All of the information in the Monarch resource is organized using ontologies, rather than free text in isolation. This means that features of the ontology can be used to assist users in search, for example, finding a disease of interest using a synonym, or using the hierarchical organization of a phenotype ontology to group annotations.

**Figure 5.**
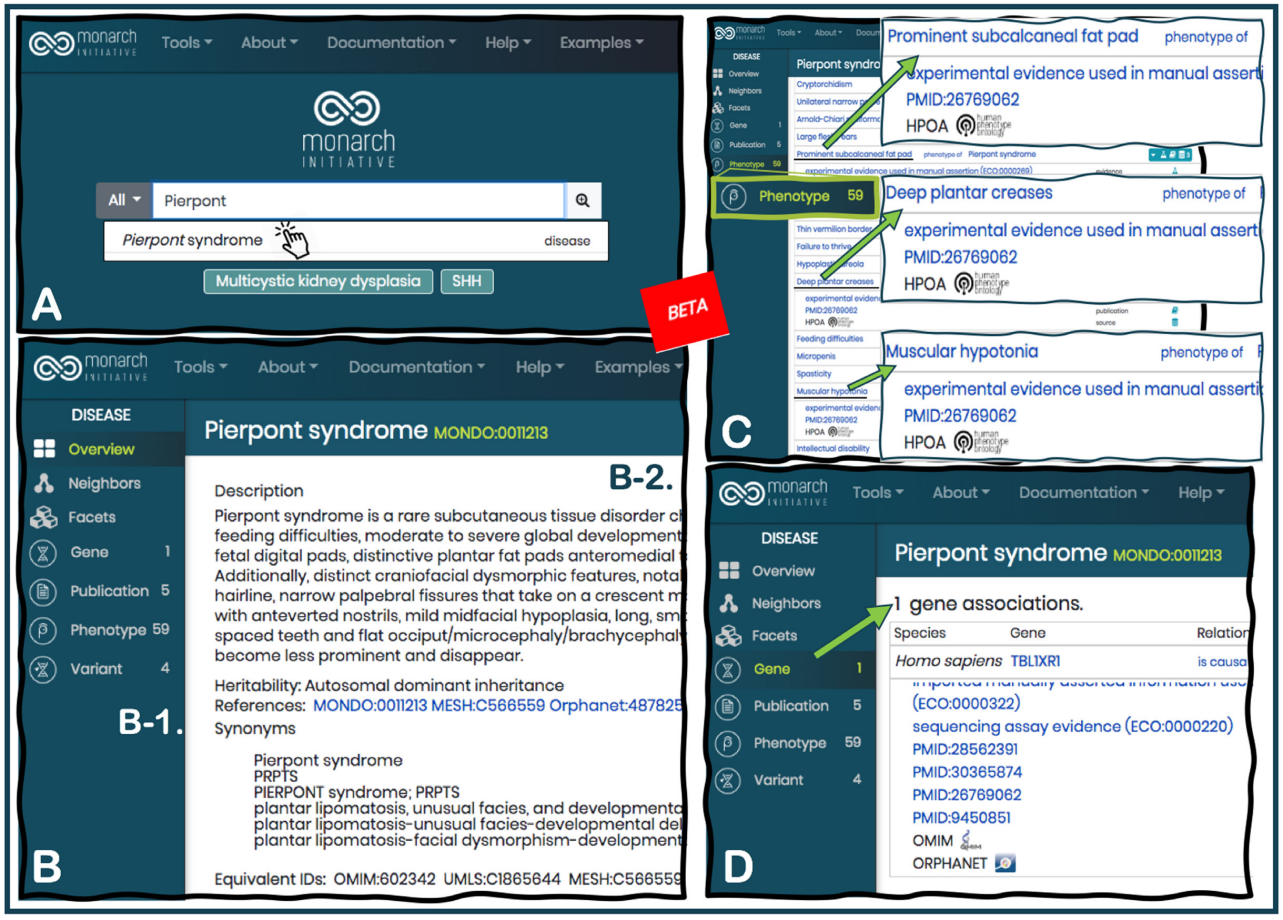
The New Monarch User Interface. A beta version of the new website is available at https://beta.monarchinitiative.org. Entering information on the ‘Search' bar, users can navigate directly to terms suggested via autocomplete, or explore more results through the results tables. In this example, a user enters only part of the name of a disease, ‘Pierpont syndrome’ (**A**). Selecting the term from the auto-complete menu, the user arrives at an overview page, which offers a summary of all available information in the integrated knowledge graph of the Monarch database (**B**). Users can explore all available data using a menu of options shown on a panel on the left (B-1), while the information is updated on the main panel on the right (B-2). In this example, the user learns that Pierpont syndrome, a rare subcutaneous tissue disorder, is characterized by phenotypes that include ‘prominent subcalcaneal fat pad' (a term in HPO, with identifier HP:0032276), ‘deep plantar creases' (HP:0001869) and ‘muscular hypotonia' (HP:0001252), among many others (**C**). Information integrated from the OMIM and Orphanet databases, as well as a number of publications, also support the association of a mutation in one gene, TBL1XR1, as the cause of Pierpont syndrome (**D**).

The main page for each phenotype, gene, or disease, the ‘Overview’ tab, offers a summary of all available information in the integrated knowledge graph of the Monarch database, and includes intuitive tools to enable users to navigate through the available data (Figure 5). In addition to finding associated terms from a variety of ontologies (for example, anatomy, function, pathway membership, orthologs, phenotypes) the updated user interface facilitates finding publications in support of all associations displayed for each term. Updated pages documenting the Patient Phenotype Curation Guidelines and the Monarch Phenotype Ontologies Project are also available from the website, as well as details about Exomiser, a Monarch tool used for prioritization of variants and candidate genes from whole exome and whole genome sequencing efforts (described below).

### Monarch Annotator

**Figure 6.**
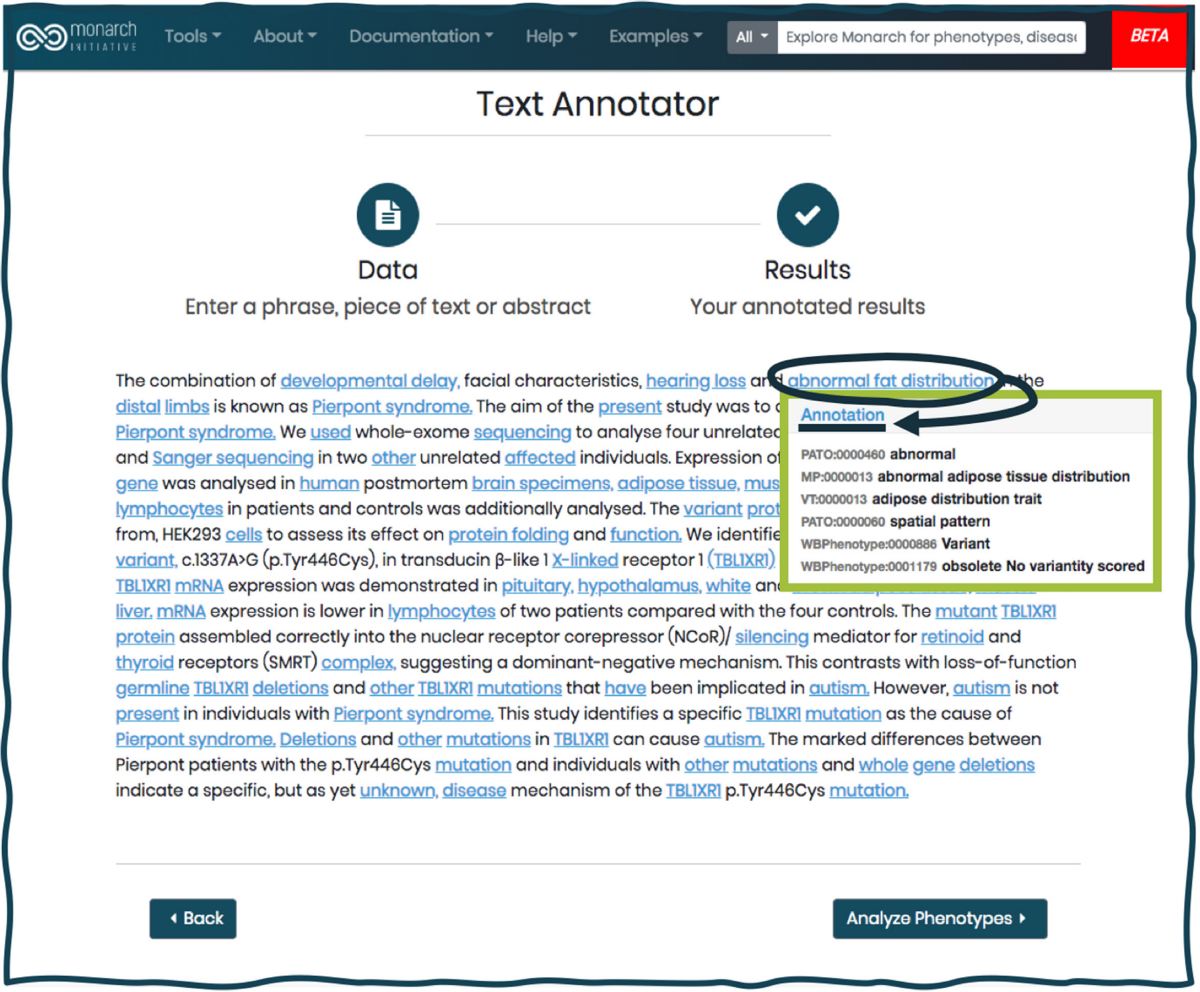
Text annotation widget on the new Monarch website. Users can supply free text and retrieve the resulting marked up text with links to terms in various ontologies. In this example, a user has entered text from a publication entitled ‘A specific mutation in TBL1XR1 causes Pierpont syndrome’ (51). The Text Annotator tool (in **beta version**) has highlighted terms identified in various ontologies, and hovering over each highlighted term offers details about the marked up annotations, in this case, ‘abnormal fat distribution.’

The new site, in addition to offering a range of options to query the Monarch database through searches, includes an updated version of our text annotation tool, which allows users to automatically mark up phenotypes, diseases, anatomical terms, genes and other entities found in text from publications via a UI (Figure 6), or using web services. This functionality was used to mine HPO (1) terms from published case reports in biocuration applications such as PhenoteFX (https://phenotefx.readthedocs.io/en/latest), which helps curators revise or create phenotype annotation records for rare disease and HpoCaseAnnotator, which is used to biocurate pathogenic variants published in scientific literature (https://hpocaseannotator.readthedocs.io).

## COMMUNITY ENGAGEMENT

Monarch's overarching goal is to galvanize translational research by developing tools, data services and analysis approaches that help scientists analyze their results in the context of genotype, phenotype and related data from a range of sources. Progress toward this goal is a virtuous loop: by building resources that benefit the community, we also incentivize others to make their tools, ontologies and data more interoperable, thereby making it even easier for a wide range of researchers to leverage Monarch's offerings. Monarch is also collaborating closely with partners such as GA4GH to develop shared standards and resources.

### The ‘Great phenotype ontology reconciliation effort’

To catalyze phenotype ontology reconciliation, we ran two workshops (Phenotype Ontologies: Traversing All The Organisms, or ‘POTATO’). The first installment, which took place at the International Conference on Biological Ontologies (ICBO) in 2018, gathered more than 40 ontology curators, developers and biomedical experts to learn about our tools for pattern-based development and to discuss discrepancies between logical definitions across various phenotype ontologies. As a result of the meeting, representatives of 14 phenotype ontologies and databases covering all major model organisms joined a common Phenotype Ontology Reconciliation Effort (https://github.com/obophenotype/upheno/wiki/Phenotype-Ontologies-Reconciliation-Effort) focused on aligning their respective ontologies by developing and using common sets of design patterns to generate logical definitions (17). The second POTATO workshop (44) was co-located with Biocuration 2019. It focused on developing strategies to deal with the limitations of the Phenotype And Trait Ontology (PATO, http://www.obofoundry.org/ontology/pato), an essential driver of inference in phenotype ontologies. Key accomplishments include the establishment of patterns and workflows for ontological alignment across many organisms; review and implementation of over 100 common design patterns across them, and the elimination of many logical and lexical mistakes (https://douroucouli.wordpress.com/2018/08/06/new-version-of-ontology-development-kit-now-with-docker-support/). Most major phenotype ontologies are now using the Ontology Development Kit (ODK, described in the ‘Ontologies’ section), which will assist with this standardization and quality control according to OBO Foundry principles (http://www.obofoundry.org/principles/fp-000-summary.html). The template-based generation of phenotype terms now allows *de-novo* generation of born-interoperable, species-specific phenotype ontologies from uPheno templates, reducing the need for manual and error-prone curation of class hierarchies. Examples of ontologies created *denovo* by this approach include the *Xenopus* Phenotype Ontology (45) and the Planarian Phenotype Ontology (http://www.obofoundry.org/ontology/planp.html).

### Exomiser

Although whole-exome and genome sequencing have revolutionized rare disease diagnostics, many cases of rare diseases remain unsolved, in part because of the difficulty of prioritizing the hundreds of candidate variants that may remain after removing those identified as common or non-pathogenic. Exomiser (46) is an automated approach developed by the Monarch Initiative to address this problem; it takes patient sequencing data and coded patient phenotypes and analyzes both, informed by a large corpus of gene-to-phenotype associations from humans and model organisms. Exomiser is being used to expedite phenotype-based variant prioritization and improve diagnosis rates in national-level programs such as the rare disease component of the UK 100 000 Genomes Project (https://www.genomicsengland.co.uk), the NIH Undiagnosed Diseases Program (4) and the European Solve-RD project (http://solve-rd.eu), individual hospitals (47) and labs and companies (congenica.com/products-and-services). It has also been used for phenotype profile matching between patients in the Matchbox tool (48) as part of the MatchMaker Exchange (MME) project (https://www.matchmakerexchange.org/) and for automated panel assignment in the Genomics England PanelAssigner tool.

### Partnerships within the Global Alliance for Genomics and Health, GA4GH

As an official Driver Project for GA4GH (https://www.ga4gh.org), the Monarch Initiative provides requirements and implementation testbeds, as well as personnel who play active leadership roles in both the *Clinical and Phenotypic Data Capture and Exchange* (CP) and the *Genomic Knowledge Standards* (GKS) Work Streams. The GKS work has leveraged several Monarch resources in its emerging standards, including the HPO, Mondo, the genotype ontology GENO and the ontology for evidence and provenance information in science, SEPIO (see ‘Ontologies’ section above). Because Monarch has been focused on the use of phenotype ontologies and terminologies in clinical and research settings, we have been significant contributors to the new information model for the exchange of clinical and genomic information, Phenopackets (https://github.com/phenopackets/phenopacket-schema). In partnership with HL7 (http://www.hl7.org/) and GA4GH, our workstream is developing a FHIR (http://hl7.org/fhir/) implementation guide for the Phenopackets schema, with the high-level goal of increasing the availability of high-quality standardized phenotypic information for genomic research and genomic medicine across the translational divide.

### NCATS Translator

The NCATS Biomedical Data Translator program (https://transltr.io) is building a structured data and machine reasoning ecosystem to address a wide range of questions about human disease posed by researchers, clinicians and patients. The three pillars of the Translator program are (i) data in the form of knowledge graphs, (ii) reusable software modules to perform computational tasks and analysis, and (iii) reasoning systems that can use data relationships and logic to provide possible answers to questions. The Monarch Initiative provides key resources to the Translator project by enabling programmatic queries via the Monarch API and by providing pre-reasoned RDF for ingest into others’ knowledge graphs. The queries retrieve specific curated and structured data on phenotypes, functional annotations and disease variants, as well as corresponding data in model organisms including gene orthologs. The Monarch data serve as the foundation for core Translator computational modules for phenotype comparison and functional similarity, as well as cross-species analytics. These modules have been used in ongoing validations of Translator performance including in a number of data-driven vignettes about chosen exemplar diseases, resulting in the first public outputs from the Translator program.

## DISCUSSION

The Monarch Initiative works closely with experts from relevant scientific data providers to ensure that the knowledge graph is useful and the data are correctly represented. This requires significant outreach with several communities of users and data providers, which takes place in the context of face-to-face workshops. Communities engaged in this way include clinicians, curators, toxicologists, exposure scientists, developmental biologists, comparative genomicists, clinical researchers, rare disease researchers and epidemiologists. These workshops provided the opportunity for experts to communicate across domains, develop use cases and provide feedback on data models. In addition to improving the data model and the ontologies, these collaborative workshops have inspired new collaborations and increased general knowledge about bio-ontologies.

The Monarch Initiative has brought together 31 data resources and made a real difference in the lives of patients (49). Monarch has been an important part of projects like GA4GH and the NCATS Translator. Future work will include development of HCLS-Compliant metadata, incorporation of additional datasets, further development of ontologies and extension of the data model to include environmental exposures. While the Monarch Initiative will continue to focus on human disease, data acquisition and model development, it will expand its scope toward a higher diversity of species and domain expert contributions.

## DATA AVAILABILITY

The Monarch platform is comprised of multiple components: user interface, data, ontologies, software tools and algorithms. The underlying data are derived from multiple external sources, the use and secondary use of which is governed by the corresponding original license for each source; as we have described in detail, this is not without complex implications (50). It is therefore not possible to provide everything under a unified license. Our integrated data corpus is available for bulk download as RDF formatted files, with subsets of this data as tab-separated value files (https://archive.monarchinitiative.org/latest). Our Neo4j database and Solr index are also publicly available in this archive. Our API provides programmatic access to associations of individual entities. General information about entities, such as definitions, synonyms and cross references, is also available at https://api.monarchinitiative.org/api. A glossary of terms and abbreviations used in this manuscript can be found at https://beta.monarchinitiative.org/glossary.
